# A Surface Acoustic Wave (SAW)-Based Lab-on-Chip for the Detection of Active α-Glycosidase

**DOI:** 10.3390/bios12111010

**Published:** 2022-11-11

**Authors:** Mariacristina Gagliardi, Matteo Agostini, Francesco Lunardelli, Alessio Miranda, Antonella Giuliana Luminare, Fabrizio Cervelli, Francesca Gambineri, Marco Cecchini

**Affiliations:** 1 NEST, Istituto Nanoscienze-CNR and Scuola Normale Superiore, Piazza San Silvestro, 56127 Pisa, Italy; 2 INTA S.R.L., Intelligent Acoustics Systems, Via Nino Pisano 14, 56122 Pisa, Italy; 3 ARCHA S.R.L., Via di Tegulaia, 10/A Ospedaletto, 56121 Pisa, Italy

**Keywords:** SAW, acoustic sensor, α-glycosidase, enzyme detection

## Abstract

Enzyme detection in liquid samples is a complex laboratory procedure, based on assays that are generally time- and cost-consuming, and require specialized personnel. Surface acoustic wave sensors can be used for this application, overcoming the cited limitations. To give our contribution, in this work we present the bottom-up development of a surface acoustic wave biosensor to detect active α-glycosidase in aqueous solutions. Our device, optimized to work at an ultra-high frequency (around 740 MHz), is functionalized with a newly synthesized probe 7-mercapto-1-eptyl-D-maltoside, bringing one maltoside terminal moiety. The probe is designed ad hoc for this application and tested in-cuvette to analyze the enzymatic conversion kinetics at different times, temperatures and enzyme concentrations. Preliminary data are used to optimize the detection protocol with the SAW device. In around 60 min, the SAW device is able to detect the enzymatic conversion of the maltoside unit into glucose in the presence of the active enzyme. We obtained successful α-glycosidase detection in the concentration range 0.15–150 U/mL, with an increasing signal in the range up to 15 U/mL. We also checked the sensor performance in the presence of an enzyme inhibitor as a control test, with a signal decrease of 80% in the presence of the inhibitor. The results demonstrate the synergic effect of our SAW Lab-on-a-Chip and probe design as a valid alternative to conventional laboratory tests.

## 1. Introduction

Gravimetric acoustic sensors are sensitive, fast, low-cost and label-free tools for the detection of analytes in gaseous or liquid samples [1]. The devices use the piezoelectric and the inverse piezoelectric effect, interconverting electrical and mechanical signals for the detection process [2]. Among gravimetric acoustic sensors, the surface acoustic wave (SAW) sensors are highly performant [3]. Selected materials and geometries determine the working principle of SAW-based sensors, providing acoustic waves with different characteristics [1]. SAW transducers can be successfully integrated in a biosensor. In a typical biosensor, a biologically active molecule (probe) decorates the surface of the sensing element exposed to the sample, and interacts with the analyte [4]. This interaction improves the performances of the sensor, and is generally fundamental to add specificity to the detection [5]. Thanks to their versatility, SAW biosensors find a variety of applications, such as in the detection of proteins [6,7,8,9,10], viruses [11,12,13,14,15,16] and bacteria [17,18,19,20,21].

Another potential application of SAW devices is related to enzyme detection and characterization. To date, the literature only reports a few studies in which the enzyme activity is gravimetrically detected by using the quartz crystal microbalance (QCM) as an acoustic device. In a typical QCM apparatus, the sensing elements are silica crystals sandwiched between two gold electrodes. The QCM sensing element exploits bulk acoustic waves (BAW) for the detection, and generally works at low frequencies (5–27 MHz). With these characteristics, QCM measures are useful for in-liquid analysis but are not as performant as the SAW devices are. With the QCM technology, the activity of some enzymes, such as proteases [22,23,24], phosphorylases [24] and DNA polymerase [25,26,27] can be studied. In this field, a particular interest is related to the detection of glycoside hydrolases, also known as glycosidases, that are enzymes for polysaccharides, such as cellulases [28,29,30] and amylases [31,32,33,34]. Glycosidase are very common enzymes in nature, and account for the catalysis of the hydrolysis of glycosidic bonds [34]. For such enzymes, detailed QCM analysis has revealed that the enzymatic reaction kinetics is not constant with the concentration of the enzyme/substrate complexes or the residues [32,33]. Moreover, the results of this approach are also valid for the discrimination of the activity of exo- or endoglycosidases [35].

In enzyme detections via QCM measures, the sensor can be functionalized by binding the substrate or the enzyme to the surface, and in some cases it has been verified that the two strategies are equivalent [36]. However, some recent studies report a different approach for the functionalization, based on the binding of nanoparticles loaded with the enzyme substrate [37,38]. Although this approach is complex and sophisticated, the presence of nanoparticles that are released from the sensor surface after the enzymatic cleavage provides the amplification of the signal. SAW sensors can provide a more sensitive detection in respect to that obtained with the QCM. The literature reports some works in which a Rayleigh SAW-based device is used for the analysis of the enzymatic reaction kinetics of trypsin [38]. Trypsin only works in an aqueous environment, where the Rayleigh SAW sensors do not work well due to the loss of the mechanical energy of the waves in the liquid phase. Thus, the SAW transducer was combined with parallel conductive electrodes, in which the conductive electrodes act as an impedance. The variations in conductivity in the liquid phase due to the enzyme activity change the electrode impedance, and this change is promptly observed as a frequency shift in the SAW transducer. The same approach is also found to be valid for the study of the enzyme activity of lipase [39,40] and acid phosphatase [41], while the combination of SAW sensors and electric devices exploiting the acoustoelectric effect, has been studied in shear horizontal SAW sensors [42]. Compared to electrical or electroacoustic sensors, a gravimetric sensor has the advantage of measuring the mass of the analyte, and the functionalization guarantees the specificity of the detection. Thus, any physical change in the analyte solution (e.g., change in conductivity of impedance) due to contaminants or side reactions do not affect the enzyme detection. To the best of our knowledge, a gravimetric SAW sensor has not yet been developed.

With the present work, we present a Lab-on-a-Chip SAW (SAW-LoC) device working at an ultra-high frequency (around 740 MHz), functionalized with a newly synthesized 7-mercapto-1-eptyl-D-maltoside, for the detection of the enzyme α-glycosidase. The maltoside units are able to be enzymatically converted in the presence of the enzyme, releasing glucose units with a related weight loss of the functionalization layer. We tested the probe with the commercial apparatus QCM with dissipation monitoring (QCM-D), in order to validate the functionalization protocol and verify the feasibility of the detection. Then, we tested our SAW-LoC device with different-concentration enzyme solutions to validate the approach. The same device was tested with samples containing the enzyme and its inhibitor acarbose, to evaluate sensor specificity.

## 2. Materials and Methods

All the materials, reagents and solvents were purchased from Sigma-Aldrich, if not otherwise stated.

### 2.1. SAW Device Fabrication and Characterization

**Figure 1:**
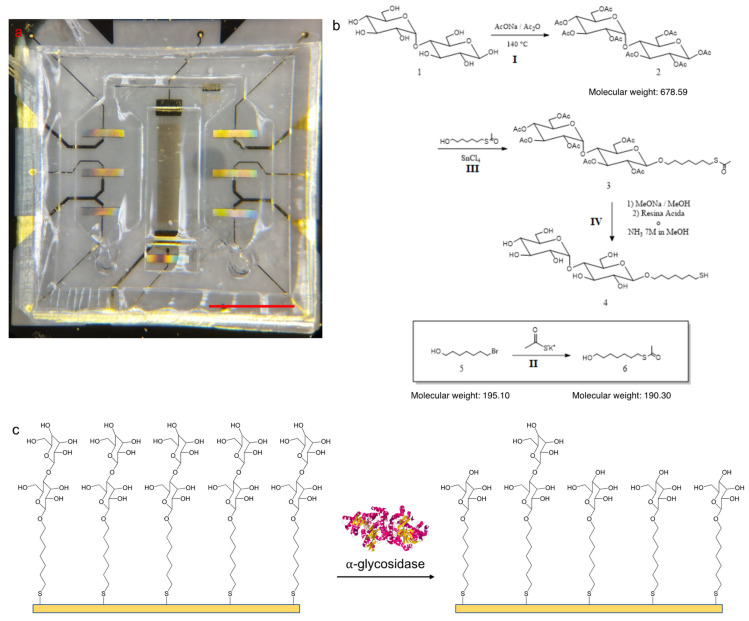
The proposed SAW-LoC biosensor: (**a**) assembled device, in which the sensors are covered with the microfluidic chamber, scale bar = 0.3 cm; (**b**) reaction scheme of the probe synthesis; (**c**) illustration of the sensor working principle: the maltoside moieties, indicated with a double blue circle, are enzymatically converted releasing glucose units.

The SAW devices (Figure 1a) used in this study were fabricated on a lithium niobate (LN) 128° YX substrate (Nano Quartz Wafer, Langenzenn, Germany) with dimensions 1 cm × 1 cm. The SAW structures present the interdigitated transducer (IDT, Newark, NJ, USA) electrodes patterned on the LN substrate previously covered with Ti/Au layer. Electrodes, consisting of seven 1-port SAW resonators designed to work at around 740 MHz, act as the sensors. One of the seven sensors, isolated from the other, is used as reference.

The Ti/Au metallization (10/100 nm) is obtained by thermal evaporation, the structure design is patterned on a layer of resist ma-N 1407 (Microresist Technology, Berlin, Germany) by laser writing (ML-3, Durham Magneto Optics, Caxton, United Kingdom, UV dose 31.6 mJ/cm^2^) and transferred to the metallic film by reactive-ion-etching (RIE, Sistec, Ar at 0.9 mbar, 50 W, 17 min). The residual Ti layer is then etched by a piranha solution (H_2_SO_4_:H_2_O_2_ 3:1 *v*/*v*, 2 min) leaving the Ti/Au only where needed.

Fabricated devices are sealed by a microfluidic chamber and a glass coverslip. The microfluidic chamber is composed of polydimethylsiloxane (PDMS, Sylgard^®^ 184, 10:1) and microchannels are patterned by curing on an SU8-2100 (Microresist Technology, Berlin, Germany) mold. There are two fluidic ports (inlet/outlet) on the microfluidic chamber, which are connected to two incubation microchambers covering the sensing resonators, and two isolated air-filled microchambers above the IDTs and the reference resonator. Inlets, outlets and two bubble trappers in the microchannels are made using a biopsy puncher. The PDMS microchannel is clamped to the chip.

The device is wire-bonded on a printed circuit board (PCB) and connected to the radiofrequency (RF) instrumentation. The midmost IDT, which is used as a mixer, is powered by a single tone RF signal at 99.7 MHz from a vector signal generator (N5181A MXG, Agilent Technologies, Santa Clara, CA, USA) followed by an RF amplifier (ZHL-5W-1, Mini-Circuits, Brooklyn, NY, USA) to have an on-chip power of 22 dBm. A vectorial network analyzer (VNA, E5071C, Agilent Technologies, Santa Clara, CA, USA) connected to an RF switch (34980A, Agilent Technologies, Santa Clara, CA, USA) allows measuring the reflected power spectrum (S11) of the single resonators at a central frequency of around 740 MHz with a span of 40 MHz at 15,001 points. An in-house software based on LabView^®^ is used to pilot the RF switch and the VNA.

### 2.2. Probe Synthesis and Characterization

7-mercapto-1-eptyl-D-maltoside was newly synthesized as sensor probe (Figure 1b). The probe and the intermediate products were evaluated by Nuclear Magnetic Resonance (NMR) analysis. Dry samples were dissolved in the appropriate solvent (~2 mg/mL) then added in a glass tube suitable for the analysis and measured with a 300 MHz Bruker Avance 300 spectrometer.

The general strategy adopted was characterized by four reaction steps:

I. Maltose (1) acetylation: this reaction is provided to protect –OH groups, the maltose is peracetylated by sodium acetate (NaOAc) and acetic anhydride (Ac_2_O), to obtain β-D-maltose-octaacetate (2) [43]. NaOAc is suspended in Ac_2_O in a molar ratio NaOAc/maltose of 1.8 and Ac_2_O/maltose of 22. The suspension is heated to reflux (T = 140–145 °C). When the refluxing starts, the heating is removed and dry maltose is added in small portions. The mixture is heated again to reflux and monitored by thin layer chromatography (Hexane/EtOAc, 4:6 *v*/*v*), after 1 h the starting reagents disappears. The reaction is stopped by adding ice water (400 mL) and vigorously stirred. The organic layer is removed by adding dichloromethane (3 times × 15 mL), then washed firstly with a saturated NaHCO_3_ solution (20 mL) and after with saturated NaCl solution (20 mL). The organic layer is dried with Na_2_SO_4_, filtered and concentrated. At the end, the residue is purified by silica gel column chromatography (Hexane/EtOAc, 4:6 *v*/*v*) to give β-D-maltose-octaacetate (2) pure, yield 91%, Rf: 0.37.

^1^H-NMR (300 MHz, CDCl3), δ/ppm: 5.73 (d,1 H, H-1), 5.41 (dd, 1 H, H-1′), 5.32 (dd, 1 H, H-3), 5.32 (dd, 1 H, H-3′), 5.06–4.94 (m, 2 H, H-4′, H-2), 4.85 (dd, 1 H, H-2′), 4.44 (dd, 1 H, H-6′), 4.23–4.18 (m, 2 H, H-6, H-6′), 4.13−4.02 (m, 2 H, H-6, H-4), 3.97–3.82 (m, 2 H, H-5, H-5′), 2.06–1.97 (m, 24 H, 8 × CH_3_). ^13^C-NMR (300 MHz, CDCl3), δ/ppm: 170.6–168.8 (8 × C=O), 95.7 (C-1′), 91.3 (C-1), 75.2 (C-3′), 73.01 (C-5), 72.5 (C-4), 71.0 (C-2), 70.03 (C-2′), 69.3 (C-3), 68.6 (C-5′), 68.0 (C-4′), 62.5 (C-6′), 61.5 (C-6), 20.6 (8 × -CH_3_).

II. Chain synthesis: the chain 7-acetylthio-1-heptanol (6) is synthetized from the 7-bromide-1-heptanol (5) by a substitution reaction with potassium thioacetate [44]. Potassium thioacetate and 7-bromide-1-heptanol (5) are added in dry tetrahydrofuran (5 mL) in molar ratio of 2:1. The mixture is firstly heated at 40 °C for 3 h and then stirred at room temperature overnight under N_2_. The product is washed with water (30 mL), and extracted with dichloromethane (3 times × 15 mL). The organic layer is washed with water and then dried with Na_2_SO_4_, filtered and concentrated. The residue is purified by silica gel column chromatography (dichloromethane/methanol, 19:1 *v*/*v*) to give 7-acetylthio-1- heptanol (6) pure, yield 74%, Rf: 0.38.

^1^H-NMR (300 MHz, CDCl3), δ/ppm: 3.6 (t, 2 H, HO-CH_2_), 2.8 (t, 2 H, S-CH_2_), 2.3 (s, 3 H, CO-CH_3_), 1.6–1.54 (m, 4 H, alkyl), 1.37–1.33 (m, 6 H, alkyl). ^13^C-NMR (300 MHz, CDCl3), δ/ppm: 196 (S- C=O), 63 (C-OH), 32.6; 30.6; 29.4; 29.07; 28.8; 28.7; 25.5 (7 × C-alkyl).

III. Maltose glycosidation: the β-D-maltose-octaacetate (2) is glycosylated by 7-acetylthio-1-heptanol (6) in dichloromethane (DCM) in the presence of tin (IV) chloride as Lewis acid catalyst, to obtain 7-acetylthio-1-heptyl-D-maltose-octaacetate (3) [45]. β-D-maltose-octaacetate (2) and 7-acetylthio-1-heptanol (6) are added in dichloromethane, under nitrogen, in molar ratio of 1:1.5. The solution is treated with 1 mL of tin (IV) tetrachloride (1 M) (molar ratio of SnCl_4_/maltose (2), 1:1), the reaction is stirred at room temperature under a nitrogen atmosphere. The reaction is monitored by thin layer chromatography (Hexane/EtOAc, 3:2 *v*/*v*) and after 1 h is stopped with a saturated NaHCO_3_ solution (10 mL). The mixture is extracted with dichloromethane (3 times × 15 mL), the organic layer is washed with saturated NaCl solution (3 times × 10 mL) and finally dried with Na_2_SO_4_, filtered and concentrated. The residue is purified by silica gel column chromatography (Hexane/EtOAc, 3:2 *v*/*v*) obtaining three fractions. The second fraction is purified by silica gel column chromatography (Hexane/EtOAc, 45:55 *v*/*v*) obtaining other three fractions, the second of them is the pure product (3), yield 4%, Rf: 0,42. (0,4)

^1^H-NMR (300 MHz, CDCl3), δ/ppm: 5.4 (d, 1 H, H-1′), 5.34 (dd, 1 H, H-3), 5.22 (dd, 1 H, H-3′), 5.10 (dd, 1 H, H-4′), 4.8–4.7 (m, 2 H, H-2, H-2′), 4.55 (2d, 1 H, H-1), 4.4 (m, 2 H, H-6, H-6′), 4.0 (m, 2 H, H-6, H-6′), 3.8 (dd, 1 H, H-4), 3.65 (ddd, 1 H, H-5′), 3.55 (ddd, 1 H, H-5), 2.8 (t, 2 H, S-CH2), 2.3 (s, 3 H, CO-CH_3_), 2.15–1.99 (m, 24 H, 8 × CH3), 1.6–1.54 (m, 4 H, alkyl), 1.36–1.28 (m, 6 H, alkyl). ^13^C-NMR (300 MHz, CDCl3), δ/ppm: 196 (S-C=O), 170.6–168.8 (7 × C=O), 100.3 (C-1), 95.6 (C-1′), 75.4 (C-3′), 72.9 (C-5), 72.7 (C-4), 72.0 (C-2), 70.8 (C-2′), 70.02 (C-3, C-5′), 69.4 (C-4′), 68.08 (C-O-), 62.9 (C-6), 61.5 (C-6′), 32.6 (-C-C-O-), 30.6; 29.4; 29.05; 28.8; 28.7; 25.7 (6 × C-alkyl), 20.6 (8 × -CH3).

IV. Deacetylation reaction: the peracetylated glycosylated maltose is deprotected by ammonia 7 N methanol to finally obtain the product, 7-mercapto-1-eptyl-D-maltoside (4) [46]. The acetylated product (3) is suspended in methanol (1 mL) and treated with 7N NH_3_/methanol (1 mL). The solution is stirred under nitrogen, the reaction is monitored by thin layer chromatography (Hexane/EtOAc, 4:6 *v*/*v*). After 6.5 h the reaction is stopped and toluene is added (2 mL), the solution is concentrated under pressure at 40 °C in presence of ethanol, the product is pure in 82% yield. In this step, NMR analysis is not necessary because the purity of the product peracetylated glycosylated (3) is previously verified, the deacetylation reaction is also verified by thin layer chromatography. The product (4) does not run on thin layer chromatography (Hexane/EtOAc, 4:6 *v*/*v*), instead the intermediate product (3) runs with Rf: 0.41.

The working principle of the maltoside-functionalized sensor (Figure 1c) is based on the evaluation of the frequency shift variation after the weight loss of the functionalization, due to the enzymatic conversion of the maltoside moieties on the probe. Thus, the synthesized probe is previously tested to evaluate the effect of time, temperature and enzyme concentration on the enzymatic conversion of the maltoside unit. The enzymatic assay was performed following one of the methods previously described in the literature [47] with a few adaptations.

An amount of 4 mL of 7-mercapto-1-eptyl-D-maltoside solution in water (5 mg/mL) was diluted with 1 mL of ethanol (80% *v*/*v*). The prepared solution was aliquoted (100 μL) in plastic tubes. Then 100 μL of enzyme solution was added in each tube. The plastic tube was then capped and maintained at the selected temperature for fixed times. Selected times were 15 min, 30 min, 60 min and 90 min. Selected temperatures were 25 °C, 40 °C and 60 °C. Selected enzyme concentrations were 0.15 U/mL, 1.5 U/mL, 15.0 U/mL and 150 U/mL, obtained in in phosphate buffer saline (PBS). Each measure was performed in triplicate. At the selected times, samples were immediately cooled and diluted by adding to the maltoside–enzyme mixture 900 μL of deionized water. Then, 100 μL of this solution was added to 200 μL of a water solution containing glucose oxidase from *Aspergillus niger* (12.5 U/mL), purpurgalin of horseradish peroxidase (2.5 U/mL) and ο-dianisidine dihydrochloride (0.1 mg/mL). The mixture was then incubated at 37 °C for 30 min under mild stirring. After 30 min, 200 μL of H_2_SO_4_ 12 N was added to the tube and gently mixed. We read the absorbance of prepared samples via UV-Vis spectroscopy (JASCO V550 spectrophotometer, JASCO Europe, Cremello, Italy) at 540 nm. As blank, we prepared samples not containing the probe and continued with the same procedure described above. As standard, we used a standard glucose water solution (0.05 mg/mL).

### 2.3. Enzyme Inhibition Test

The enzyme activity was tested with its inhibitor acarbose (TCI Europe, Zwijndrecht, Belgium) measuring samples prepared with different enzyme and acarbose concentrations. Selected concentrations of the enzyme were 0.15 U/mL, 1.5 U/mL, 15.0 U/mL and 150 U/mL, solutions were prepared in water. For acarbose, used concentrations were 0.1 mg/mL, 1.0 mg/mL, 2.5 mg/mL, 5.0 mg/mL and 10.0 mg/mL, solutions were prepared in a 67 mM potassium phosphate buffer, pH 6.8.

Samples were prepared by adding to a plastic vial, 500 μL of potassium phosphate buffer, 20 μL of a 3 mM glutathione reduced solubilized in water, 20 μL of the enzyme solution and 20 μL of the inhibitor solution. The mixture was gently mixed and equilibrated at 37 °C, then 50 μL of 10 mM p-nitrophenyl α-D-glycoside solution in water (α-glycosidase substrate) was added. This solution was gently mixed and incubated at 37 °C for 20 min after incubation, 200 μL of the prepared sample was added to 800 μL of 100 mM sodium carbonate solution in water and gently mixed. Absorbances of such samples were read by UV-Vis spectrometer at 400 nm.

Measures were performed in triplicate. As reference, the activity of the non-inhibited enzyme and the absorbance of solutions not containing the enzyme were measured.

### 2.4. Optimization of Surface Functionalization via QCM-D and Sensor Functionalization

All the QCM-D E4 model, Q-Sense AB (Biolin, Västra Frölunda Sweden) measures were performed with polished AT-cut quartz crystals (gold electrodes, fundamental resonance frequency *f_0_* = 5 MHz, diameter = 14 mm, thickness = 100 nm) in static mode (stop flow). Crystals were treated, prior to use, with plasma oxygen (Femto Diener, 10 min, power 100 W), washed with a 5:1:1 solution of water, ammonia (32% *v*/*v*) and oxygen peroxide (25% *v*/*v*) at 75 °C for 15 min, rinsed with water and after with isopropanol, finally treated with plasma oxygen again (10 min, power 100 W). The fluidic cells were thermostatted at 25 °C. The apparatus allows simultaneous recording of the crystal resonance frequency shift (Δ*f*) and energy dissipation (Δ*D*) for up to 13 overtones, by exciting the fundamental resonance frequency of the crystal. In this work, we chose the overtones 3, 5, 7 and 9 for data analysis as the most sensitive and stable among the entire dataset. Measures were performed on four sensors for each analyzed sample.

The thiolate probe was directly attached to the Au electrode surface via a thiol-Au chemistry. The functionalization solution was prepared dissolving the probe 7-mercapto-1-eptyl-D-maltoside in water (1 mg/mL) with added dithiothreitol (DTT) in concentration 3.5x mol/mol in respect to the free thiols. QCM-D sensors were first rinsed with water and stabilized waiting for a sufficient time until the acquired signals did not show drifts (Δ*F* < 1 Hz over 30 min). When sensors were stable, the functionalization solutions are injected in the QCM-D chamber and data were acquired for 90 min. Δ*D* values, which were strictly related to the mechanical behaviour of the functionalization layer, were checked for the application of the Sauerbrey model. In this work, the criterium used to consider the Sauerbrey model valid was Δ*D* < 2.0 × 10^−6^ [48].

The functionalization procedure was then transferred on the SAW-LoC device. Functionalizations, in this case, were obtained by placing droplets (2 µL) of the functionalization solution on the resonator surface. During functionalization, the devices were maintained under a water-saturated atmosphere to prevent droplet evaporation. At the end of the functionalization, the devices were rinsed by dipping the chip into deionized water, and gently dried under nitrogen flux.

### 2.5. Sample Detection

The sample to detect was the enzyme α-glycosidase from *Bacillus stearothermophilus* (Megazyme Ltd., Wicklow, Ireland). Different-concentration solutions containing the enzyme α-glycosidase were injected in the microfluidic chamber of the QCM-D and of the sensor and maintained in incubation for 60 min. Selected enzyme concentrations in PBS were 0.15 U/mL, 1.5 U/mL, 15.0 U/mL and 150 U/mL. At the end of the incubation, QCM-D sensors were rinsed with water and signals were acquired for 10 min, while the SAW-LoC sensors were rinsed with distilled water and dried under a mild nitrogen flux.

### 2.6. Data Analysis

For QCM-D measures, we performed at least four measures for each analyzed sample. Δ*F* and Δ*D* were continuously recorded during all the different steps of the experiments: sensor conditioning, functionalization, detection and the related rinsing. Areal mass (ng cm^−2^) values were calculated according to the Sauerbrey equation (Equation (1)):

(1) $$\Delta m\;=\;-C\cdot \frac{\Delta F}{n}$$

where Δ*m* (ng cm^−2^) is the mass variation of the crystal in respect to the baseline, *C* is the mass sensitivity constant (17.7 ng cm^−2^ Hz^−1^, for 5 MHz crystal quartz), Δ*F* is the frequency shift after rinsing and *n* is the overtone number.

For the SAW-LoC device, we acquired at least five spectra for each resonator before and after the sample detection. The resonance frequency shift of each resonator was calculated by using an algorithm based on the cross-correlation among the spectra. The final frequency shift from the sample analysis with the SAW-LoC device was calculated as the average and the standard error of the signal from the six biosensors before the sample injection and after the rinsing, corrected by subtracting the signal from the reference sensor. The statistical analysis of data was performed with the Mann–Whitney U-test.

In boxplots, the box indicates the data range from the first to the third quartile, the inner line through the box is the median value, and the whiskers delimitate the overall data range. Through the text, data are reported as mean values of independent experiments, error bars indicate standard errors.

## 3. Results

### 3.1. Enzymatic Conversion of Maltoside Probe and Inhibition Assay

**Figure 2:**
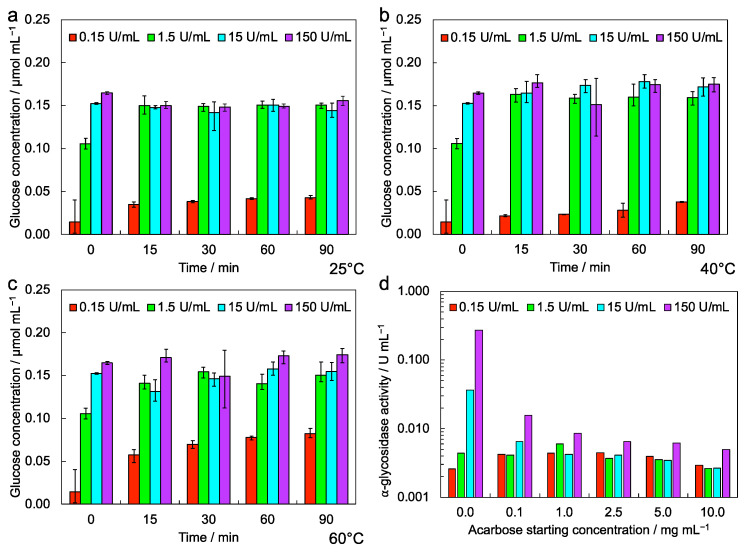
In-cuvette characterization of probe and enzyme: enzymatic conversion of the probe at (**a**) 25 °C, (**b**) 40 °C and (**c**) 60 °C by varying the enzyme concentration; (**d**) enzyme activity of α-glycosidase in the presence of its inhibitor acarbose in different concentrations.

We evaluated the glucose concentration in samples containing the probe after the enzymatic cleavage of the maltoside unit (Figure 2a–c). The glucose concentration reached the maximum value (0.15 μmol/mL) at all the tested temperatures in samples treated with α-glycosidase solutions with starting concentrations of 1.5 U/mL, 15 U/mL and 150 U/mL. The enzymatic conversion in samples treated with α-glycosidase solutions with starting concentrations of 0.15 U/mL was very low at 25 °C, and showed only a mild increase with the increasing temperature.

The enzyme activity measured in inhibition tests (Figure 2d), performed by adding acarbose to enzyme samples, was significantly lowered in all cases, and in particular for the higher enzyme concentrations, in respect to that calculated in samples not containing acarbose.

From this preliminary analysis, we selected 25 °C as the working temperature for the SAW-LoC measures, and the concentration of acarbose equal to 2.5 mg/mL for control measures.

### 3.2. QCM-D Measures

**Figure 3:**
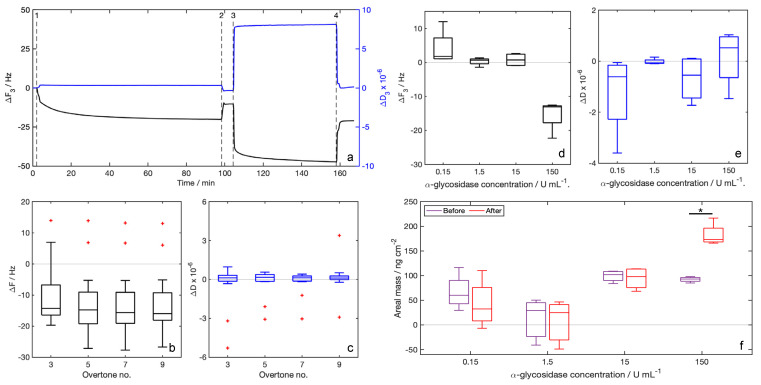
Results of QCM-D measures: (**a**) traces acquired during the experiments (black: Δ*F*, blue: Δ*D*) referred to the third overtone, events: (1) functionalization solution injection, (2) rinsing with water, (3) enzyme solution (150 U/mL) injection, (4) rinsing with water, reported traces are related to the experiment with enzyme concentration of 150 U/mL; (**b**) Δ*F* measured after rinsing with water for the functionalization for the selected overtones (*n* = 16 for each overtone); (**c**) Δ*D* measured after rinsing with water for the functionalization for the selected overtones (*n* = 16 for each overtone); (**d**) Δ*F_3_* measured after rinsing with water for the injection of different enzyme concentrations (*n* = 4 for each enzyme concentration), values are calculated as Δ*F* between events 4 and 3; (**e**) Δ*D_3_* measured after rinsing with water for the injection of different enzyme concentrations (*n* = 4 for each enzyme concentration), values are calculated as Δ*D* between events 4 and 3; (**f**) probe areal masses (ng cm^−2^) calculated from Δ*F_3_* using the Sauerbrey equation (Equation (1)). For this calculation, experiments giving outliers, indicated by the red crosses in plots b and c, after the functionalization, are eliminated (*n* = 4 in all cases except for the experiment with enzyme concentration 1.5 U/mL, for which *n* = 3), values are calculated as Δ*F* between events 4 and 1. Statistical tests are performed comparing the frequency shift due to functionalization and to samples, * indicates *p* < 0.1, obtained from a Mann–Whitney U-test.

Traces of the whole experiment clearly highlight all the events occurring during the experiment. Referring to Figure 3a, the event indicated with 1 (injection of the functionalization solution) is followed by a decrease in the resonance frequency, while dissipation slightly increases. Both strong and labile interactions between the sensor gold electrode and the probe provide a mass loading and, thus, negative frequency shifts. After event 2 (rinsing with water), the frequency increases and the dissipation decreases because molecules attached with labile interactions to the sensors are washed off. Shifts after rinsing are considered to be related only to the probe molecules covalently attached to the sensor surface. The enzyme solution is injected at event 3. After the injection, frequency and dissipation trends depend on the enzyme concentration. Figure 3a reports the trace of the experiment performed with the higher enzyme concentration (150 U/mL), for which the frequency decreases and the dissipation increases after the enzyme solution injection. In all other cases, trends follow the opposite behavior (traces not shown). In the last part of the experiment, at event 4 (rinsing with water), the enzyme molecules and the glucose moieties formed are washed off and the final values of frequency and dissipation are obtained.

For the characterization of the functionalization layer, we can refer to the median values of Δ*F*. The median Δ*F* values obtained after the functionalization step with the synthesized 7-mercapto-1-eptyl-D-maltoside (Figure 3b) are around −16 Hz, without any dependency on the overtone number. Values of Δ*D* related to the functionalization layer (Figure 3c) are all close to zero. Δ*F* net values measured after rinsing (Figure 3d), calculated using the value after functionalization as the baseline, are positive in all experiments except for that with the higher enzyme concentration (150 U/mL), and the opposite trend is registered for net Δ*D* (Figure 3e). Probe areal masses calculated with the Sauerbrey equation (Equation (1)) before the injection of the enzyme solution are in all cases higher than those calculated after the injection of the enzyme solution except for the higher enzyme concentration (Figure 3f).

### 3.3. SAW Device Measures

**Figure 4:**
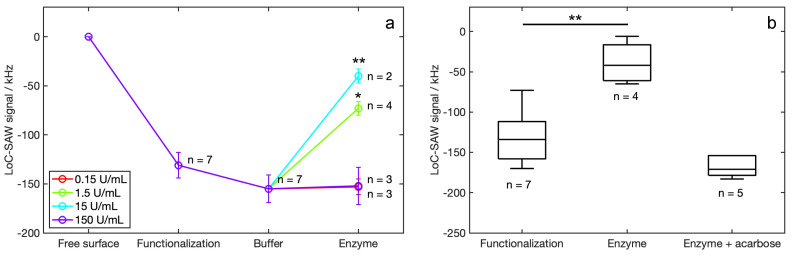
Measures performed with the SAW-LoC device: (**a**) characteristic curve obtained analyzing samples containing α-glycosidase in concentrations ranging from 0.15 to 150 U/mL; (**b**) frequency shifts measured analyzing samples containing α-glycosidase (15 U/mL) and its inhibitor acarbose (2.5 mg/mL), compared to those obtained after the functionalization and the injection of samples containing only the enzyme (15 U/mL); *n* indicates the number of sensors used for each analysis; statistical tests are performed comparing the frequency shift from functionalization and from samples, * indicates *p* < 0.1, while ** indicates *p* < 0.01, obtained from a Mann–Whitney U-test.

Readouts acquired with the SAW-LoC sensor after the functionalization with the synthesized probe and the injection of different-concentration enzyme solutions (Figure 4a) indicate negative frequency shifts in all cases. The signal related to the sensor functionalization result was −131 kHz ± 13 kHz. We also registered a small signal of −24 kHz ± 13 kHz related to the PBS used as enzyme buffer. The sum of those signals, equal to −158 kHz ± 14 kHz, was considered the baseline for the following enzyme detection experiments. The frequency shifts read after the injection of the enzyme solutions with the higher (150 U/mL) and the lower (0.15 U/mL) concentrations were negligible, while significant positive net frequency shifts were read after the injection of the enzyme solutions with intermediate concentrations (1.5 U/mL and 15 U/mL).

Control experiments obtained the injection over the functionalized sensor surface of a solution containing the enzyme (15 U/mL) and its inhibitor acarbose (2.5 mg/mL). The final shifts (Figure 4b), calculated using the signal acquired for the free surface as a baseline, were −42 ± 10 kHz and −171 ± 5 kHz.

## 4. Discussion

In-cuvette tests performed to evaluate the enzymatic conversion of the probe (Figure 2a–c) indicate a full enzymatic conversion of the maltoside unit in samples treated with α-glycosidase solutions with concentrations of 1.5 U/mL, 15 U/mL and 150 U/mL. In such cases, the probe results are completely cleaved and the sensor signal related to the probe weight loss should be maximized. The in-cuvette test performed to evaluate the enzyme activity inhibition in the presence of acarbose (Figure 2d) indicates, as a general rule, that increasing concentrations of acarbose lead to a lower enzyme activity. The registered activity in samples prepared with α-glycosidase solution with the higher starting concentration (150 U/mL) and the higher starting acarbose concentration (10 mg/mL) results in around 2% of that measured in samples with the same enzyme concentrations but without acarbose. Important activity decreases are also registered in samples with the higher inhibitor concentration and the α-glycosidase starting concentrations of 15 U/mL and 1.5 U/mL, resulting in ~7% and ~60%, respectively, compared with those measured without acarbose. Samples containing the lower enzyme concentration (0.15 U/mL) do not show a measurable activity decrease. On the basis of the in-cuvette tests, a good pair for measures with sensors are 15 U/mL for the α-glycosidase solution and 2.5 mg/mL for the acarbose solution.

QCM-D measures were preliminarily performed to setup and verify the sensor functionalization procedure and the feasibility of enzyme detection. First, we performed the functionalization experiments to verify the probe attachment and the repeatability of the functionalization. We collected data from 16 independent sensors. QCM-D results for the sensor functionalization with the newly synthesized probe gave, in all cases, negative median Δ*F* values after the rinsing with water (Figure 3b), in respect to the baseline (water). Negative frequency shifts in acoustic gravimetric sensors are conventionally related to a mass loading effect, and thus to a successful deposition of molecules over the sensor surface. In our case, the mass loading effect is related to the binding of the probe to the gold surface of the electrode. Median frequency shifts measured were around −15 Hz without any dependence on the overtone number. It ensures that the proposed procedure for sensor functionalization is working and reliable. Median values of energy dissipation shift Δ*D* (Figure 3c) were all lower than 2 × 10^−6^, thus, the Sauerbrey equation (Equation (1)) is valid and we can calculate the amount of probe linked to the sensor surface. The probe areal mass results, 93 ± 4 ng/cm^2^, calculated for the third overtone, correspond to 197 ± 9 pmol/cm^2^.

After the injection of different-concentration α-glycosidase solutions and the rinsing with water, we registered a negative frequency shift for the higher enzyme concentration, while there were slightly positive values in all other cases (Figure 3d). The negative Δ*F* measured after the detection of the more concentrated enzyme sample can be explained by hypothesizing about the deposition of the enzyme over the surface due to the high concentration. The enzyme deposition leads to a mass loading effect, and then to a negative frequency shift. The positive Δ*F* registered can be related to the enzymatic conversion of the probe, which loses a glucose unit. Additionally in these measures, the dissipation shift (Figure 3e) is lower than the limit value for the application of the Sauerbrey equation; thus, we can calculate the amount of mass loss after the enzymatic conversion. The loss in weight of the functionalization layer (Figure 3f) is small but detectable. The loss of areal mass is 10 ± 8 ng/cm^2^ for the experiment performed by injecting a 0.15 U/mL α-glycosidase solution, and 4 ± 2 ng/cm^2^ for the experiment with α-glycosidase concentrations of 1.5 U/mL and 15 U/mL. Reported weight losses correspond to 58 ± 43 pmol/cm^2^ (enzyme concentration 0.15 U/mL), 22 ± 10 pmol/cm^2^ (1.5 U/mL) and 24 ± 16 pmol/cm^2^ (1.5 U/mL), calculated in respect to the glucose units. Such results indicate an enzymatic conversion of around one-third of the probe molecules over the sensor surface for the lower enzyme concentration in the sample, and around one out of nine for samples with enzyme concentrations of 1.5 U/mL and 15 U/mL. However, the QCM-D results are not in line with those obtained in the cuvette experiments. These differences, as well as the variability in the results, indicate that these measurements require better performing apparatus, such as our SAW-LoC sensor that operates at ultra-high frequencies.

The frequency shift measured after the functionalization in the SAW-LoC sensor was negative in respect of the baseline (free surface), confirming the occurred chemical functionalization of the surface. After sample injection, we did not measure any frequency shift for the higher and the lower enzyme concentrations. The low frequency shift attributed to the α-glycosidase sample with concentration 0.15 U/mL was due to the too low enzyme activity, which was not sufficient to provide the enzymatic conversion of the probe, as verified with in-cuvette experiments. The low frequency shift given by the higher α-glycosidase sample (150 U/mL) can be explained with the occurrence of a significant mass loading due to the adhesion of the enzyme over the functionalized sensor surface, as already registered in QCM-D measures. Significant frequency increases, in respect to the baseline acquired after the functionalization, were measured after the injection of enzyme solutions with concentrations of 1.5 U/mL and 15 U/mL, indicating the occurred enzymatic conversion of the probe. We measured a lower shift for the concentration 1.5 U/mL in respect to that obtained with the solution with concentration 15 U/mL, which is in line with results from in-cuvette experiments. Our sensor allows analyte detections to be obtained in around 60 min, without any preliminary sample preparation or training for the operator. Conventional laboratory techniques are generally based on enzyme assays. Enzyme assays involve the use of several reagents and a synthetic substrate for the enzyme. The synthetic substrate is commonly modified to release a moiety, detectable via fluorescence spectroscopy or that become colored to be analyzed by UV-Vis spectroscopy after an appropriate treatment. Thus, enzyme assays are often long, complex and expensive. Moreover, the procedures should be performed by specialized personnel, equipped by specific instruments. Conversely, a SAW-based sensor for enzyme detection gives the results rapidly, reducing costs related to the sample treatment and handling, and can be performed out of a laboratory. Moreover, the proposed SAW sensor shows some advantages in respect to other detection techniques. As an example, optical devices for enzyme detection are commonly based on the available spectroscopic techniques. Transduction systems in optical sensors are expensive and difficult to miniaturize, making portable devices hard to obtain. In addition, some highly-performant optical techniques are not useful for all enzyme detections. In our case, the detection of α-glycosidase involves the production of glucose units from the substrate maltose. Optical techniques such as surface plasmon resonance, based on a local change in the refractive index upon the interaction of a metal nanostructure with the analyte, can give erroneous results due to the aggregation of the nanostructure used in the presence of glucose [49]. Other optical techniques need labels. Generally, such labels are synthetic fluorescent markers, such as dyes or quantum dots, that can exhibit photobleaching or ageing with time, making the detection less performant over time [50].

Control experiments were performed on the SAW-LoC sensor by injecting samples containing the active α-glycosidase enzyme together with its inhibitor acarbose. In this experiment we recorded an almost null signal, equal to −38 kHz ± 26 kHz, in respect to the signal acquired after the functionalization. This characterization confirms that the probe weight loss is only related to the enzymatic conversion of the maltoside units.

## 5. Conclusions

Our SAW-LoC device, functionalized with the newly synthesized probe, showed a high potential for the detection of the enzyme α-glycosidase. The synthesized probe, carrying a maltoside terminal moiety, was shown to undergo the enzymatic conversion of the maltoside in the presence of the active enzyme, proving suitable for the proposed detection. QCM-D measures, used to optimize and verify the functionalization of the sensor surface, confirmed that the proposed functionalization protocol is suitable for the functionalization. On the other hand, sample detection with QCM-D did not work for a quantitative analysis of the enzyme, but indicated that the strategy could work on more performant sensors. SAW-LoC measures confirmed that the proposed strategy is suitable for the detection of the active enzyme in the samples, providing a positive frequency shift that is related to a loss in weight of the functionalization layer. Unlike the commercial QCM-D apparatus, the SAW-LoC device demonstrated a potential for the quantitative measure of the enzyme, after the appropriate calibration in different working conditions. As further characterization, we will study the enzyme conversion at different times and temperatures, in order to check the sensor’s performance in different working conditions.

With this work, we demonstrated the feasibility of α-glycosidase detection by means of a gravimetric SAW device working under an ultra-high frequency regime. As a future development, we will apply our technology to the detection of different enzymes, in order to provide a tool for rapid detection as a cheap and fast alternative to the conventional laboratory techniques, such as UV-Vis spectroscopy or fluorescence spectroscopy, and to avoid the involvement of complex instruments and trained personnel.

## Data Availability

Datasets generated during the study are freely available on the DRYAD platform (www.datadryad.org, accessed on 11 October 2022), at the following link: https://doi.org/10.5061/dryad.tmpg4f52k.
